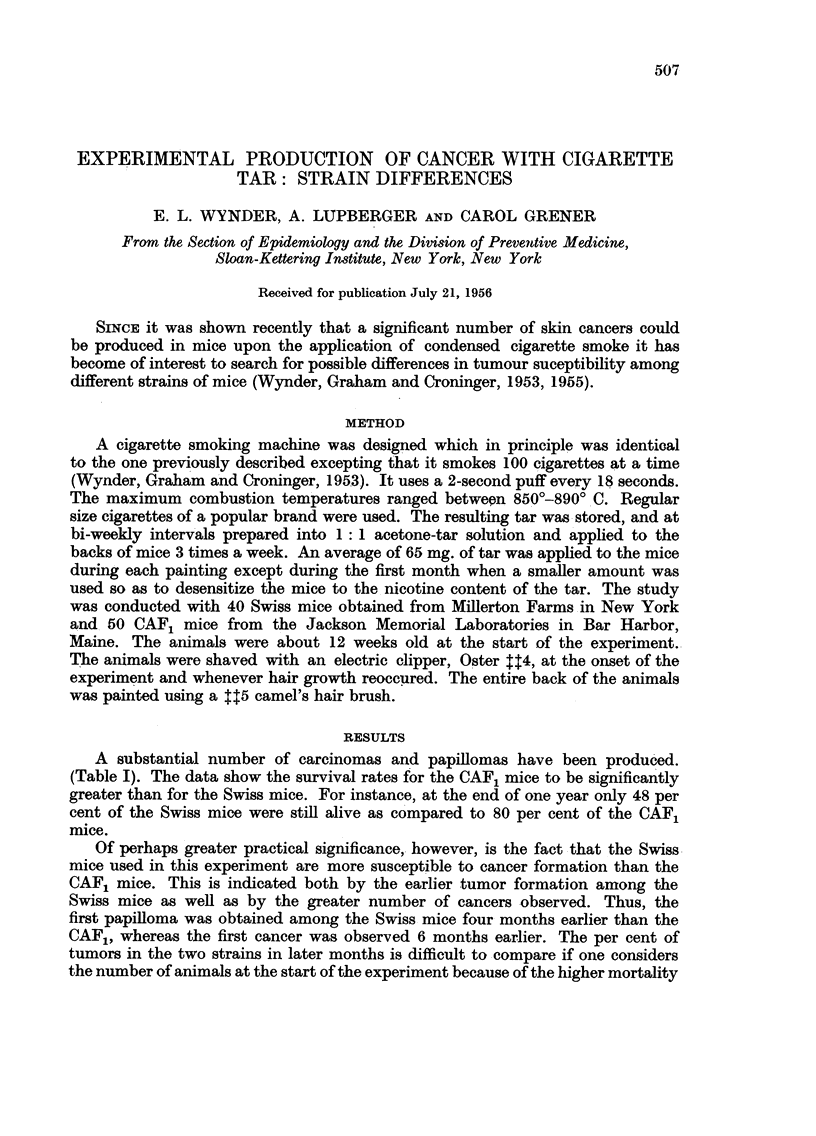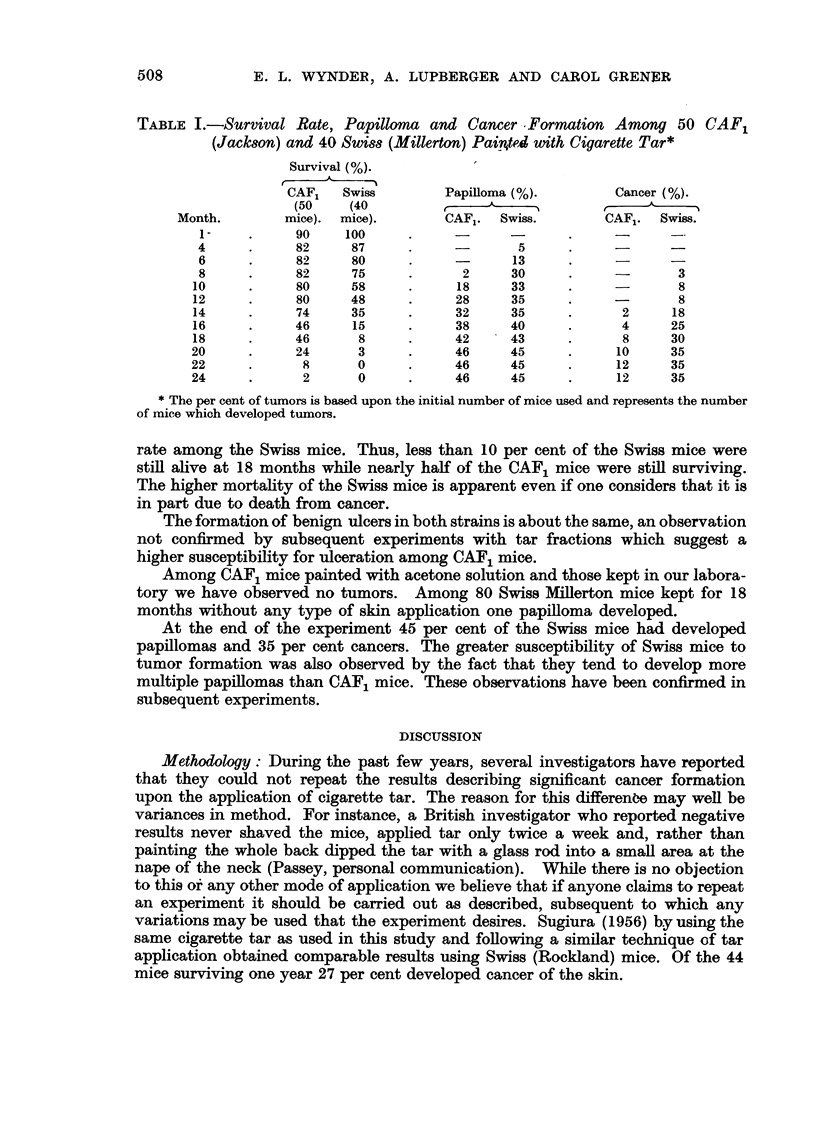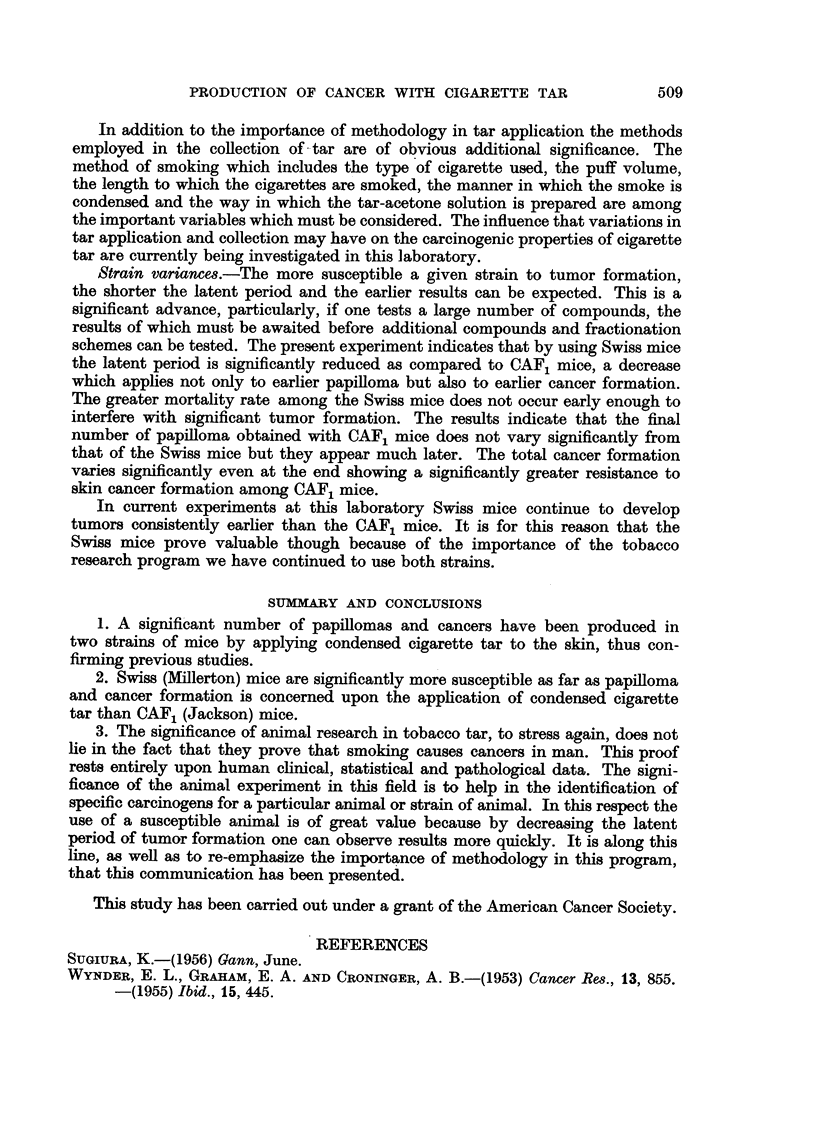# Experimental Production of Cancer with Cigarette Tar: Strain Differences

**DOI:** 10.1038/bjc.1956.58

**Published:** 1956-09

**Authors:** E. L. Wynder, A. Lupberger, Carol Grener


### EXPERIMENTAL PRODUCTION OF CANCER WITH CIGARETTE TAR : STRAIN DIFFERENCES

E. L. WYNDER, A. LUPBERGER AND CAROL GRENER

*From the Section of Epidemiology and the Division of Preventive Medicine, Sloan-Kettering Institute, New York, New York*

Received for publication July 21, 1956

SINCE it was shown recently that a significant number of skin cancers could be produced in mice upon the application of condensed cigarette smoke it has become of interest to search for possible differences in tumour suceptibility among different strains of mice (Wynder, Graham and Croninger, 1953, 1955).

### METHOD

A cigarette smoking machine was designed which in principle was identical to the one previously described excepting that it smokes 100 cigarettes at a time (Wynder, Graham and Croninger, 1953). It uses a 2-second puff every 18 seconds. The maximum combustion temperatures ranged between 850°–890° C. Regular size cigarettes of a popular brand were used. The resulting tar was stored, and at bi-weekly intervals prepared into 1 : 1 acetone-tar solution and applied to the backs of mice 3 times a week. An average of 65 mg. of tar was applied to the mice during each painting except during the first month when a smaller amount was used so as to desensitize the mice to the nicotine content of the tar. The study was conducted with 40 Swiss mice obtained from Millerton Farms in New York and 50 $CAF_1$ mice from the Jackson Memorial Laboratories in Bar Harbor, Maine. The animals were about 12 weeks old at the start of the experiment. The animals were shaved with an electric clipper, Oster ‡‡4, at the onset of the experiment and whenever hair growth reoccured. The entire back of the animals was painted using a ‡‡5 camel's hair brush.

### RESULTS

A substantial number of carcinomas and papillomas have been produced. (Table I). The data show the survival rates for the $CAF_1$ mice to be significantly greater than for the Swiss mice. For instance, at the end of one year only 48 per cent of the Swiss mice were still alive as compared to 80 per cent of the $CAF_1$ mice.

Of perhaps greater practical significance, however, is the fact that the Swiss mice used in this experiment are more susceptible to cancer formation than the $CAF_1$ mice. This is indicated both by the earlier tumor formation among the Swiss mice as well as by the greater number of cancers observed. Thus, the first papilloma was obtained among the Swiss mice four months earlier than the $CAF_1$, whereas the first cancer was observed 6 months earlier. The per cent of tumors in the two strains in later months is difficult to compare if one considers the number of animals at the start of the experiment because of the higher mortality

TABLE I.—*Survival Rate, Papilloma and Cancer Formation Among* 50 $CAF_1$ *(Jackson) and* 40 *Swiss (Millerton) Painted with Cigarette Tar**

| Month. | Survival (%). $CAF_1$ (50 mice). | Survival (%). Swiss (40 mice). | Papilloma (%). $CAF_1$. | Papilloma (%). Swiss. | Cancer (%). $CAF_1$. | Cancer (%). Swiss. |
|---|---|---|---|---|---|---|
| 1 | 90 | 100 | — | — | — | — |
| 4 | 82 | 87 | — | 5 | — | — |
| 6 | 82 | 80 | — | 13 | — | — |
| 8 | 82 | 75 | 2 | 30 | — | 3 |
| 10 | 80 | 58 | 18 | 33 | — | 8 |
| 12 | 80 | 48 | 28 | 35 | — | 8 |
| 14 | 74 | 35 | 32 | 35 | 2 | 18 |
| 16 | 46 | 15 | 38 | 40 | 4 | 25 |
| 18 | 46 | 8 | 42 | 43 | 8 | 30 |
| 20 | 24 | 3 | 46 | 45 | 10 | 35 |
| 22 | 8 | 0 | 46 | 45 | 12 | 35 |
| 24 | 2 | 0 | 46 | 45 | 12 | 35 |

* The per cent of tumors is based upon the initial number of mice used and represents the number of mice which developed tumors.

rate among the Swiss mice. Thus, less than 10 per cent of the Swiss mice were still alive at 18 months while nearly half of the $CAF_1$ mice were still surviving. The higher mortality of the Swiss mice is apparent even if one considers that it is in part due to death from cancer.

The formation of benign ulcers in both strains is about the same, an observation not confirmed by subsequent experiments with tar fractions which suggest a higher susceptibility for ulceration among $CAF_1$ mice.

Among $CAF_1$ mice painted with acetone solution and those kept in our laboratory we have observed no tumors. Among 80 Swiss Millerton mice kept for 18 months without any type of skin application one papilloma developed.

At the end of the experiment 45 per cent of the Swiss mice had developed papillomas and 35 per cent cancers. The greater susceptibility of Swiss mice to tumor formation was also observed by the fact that they tend to develop more multiple papillomas than $CAF_1$ mice. These observations have been confirmed in subsequent experiments.

### DISCUSSION

*Methodology :* During the past few years, several investigators have reported that they could not repeat the results describing significant cancer formation upon the application of cigarette tar. The reason for this difference may well be variances in method. For instance, a British investigator who reported negative results never shaved the mice, applied tar only twice a week and, rather than painting the whole back dipped the tar with a glass rod into a small area at the nape of the neck (Passey, personal communication). While there is no objection to this or any other mode of application we believe that if anyone claims to repeat an experiment it should be carried out as described, subsequent to which any variations may be used that the experiment desires. Sugiura (1956) by using the same cigarette tar as used in this study and following a similar technique of tar application obtained comparable results using Swiss (Rockland) mice. Of the 44 mice surviving one year 27 per cent developed cancer of the skin.

In addition to the importance of methodology in tar application the methods employed in the collection of tar are of obvious additional significance. The method of smoking which includes the type of cigarette used, the puff volume, the length to which the cigarettes are smoked, the manner in which the smoke is condensed and the way in which the tar-acetone solution is prepared are among the important variables which must be considered. The influence that variations in tar application and collection may have on the carcinogenic properties of cigarette tar are currently being investigated in this laboratory.

*Strain variances.*—The more susceptible a given strain to tumor formation, the shorter the latent period and the earlier results can be expected. This is a significant advance, particularly, if one tests a large number of compounds, the results of which must be awaited before additional compounds and fractionation schemes can be tested. The present experiment indicates that by using Swiss mice the latent period is significantly reduced as compared to $CAF_1$ mice, a decrease which applies not only to earlier papilloma but also to earlier cancer formation. The greater mortality rate among the Swiss mice does not occur early enough to interfere with significant tumor formation. The results indicate that the final number of papilloma obtained with $CAF_1$ mice does not vary significantly from that of the Swiss mice but they appear much later. The total cancer formation varies significantly even at the end showing a significantly greater resistance to skin cancer formation among $CAF_1$ mice.

In current experiments at this laboratory Swiss mice continue to develop tumors consistently earlier than the $CAF_1$ mice. It is for this reason that the Swiss mice prove valuable though because of the importance of the tobacco research program we have continued to use both strains.

### SUMMARY AND CONCLUSIONS

1. A significant number of papillomas and cancers have been produced in two strains of mice by applying condensed cigarette tar to the skin, thus confirming previous studies.
2. Swiss (Millerton) mice are significantly more susceptible as far as papilloma and cancer formation is concerned upon the application of condensed cigarette tar than $CAF_1$ (Jackson) mice.
3. The significance of animal research in tobacco tar, to stress again, does not lie in the fact that they prove that smoking causes cancers in man. This proof rests entirely upon human clinical, statistical and pathological data. The significance of the animal experiment in this field is to help in the identification of specific carcinogens for a particular animal or strain of animal. In this respect the use of a susceptible animal is of great value because by decreasing the latent period of tumor formation one can observe results more quickly. It is along this line, as well as to re-emphasize the importance of methodology in this program, that this communication has been presented.

This study has been carried out under a grant of the American Cancer Society.

### REFERENCES

SUGIURA, K.—(1956) *Gann*, June.
WYNDER, E. L., GRAHAM, E. A. AND CRONINGER, A. B.—(1953) *Cancer Res.*, **13**, 855.
—(1955) *Ibid.*, **15**, 445.